# COVID-19 and the Taliban-shadowed intensification of the neglected fate of viral hepatitis in Afghanistan

**DOI:** 10.1016/j.amsu.2022.104971

**Published:** 2022-11-17

**Authors:** Jawad Amini, Nooria Mohammady, Arefa Mohammadi, Shekiba Madadi, Shamim Arif, Faridullah Omary, Akihiko Ozaki, Binaya Sapkota, Sunil Shrestha, Sayed Hamid Mousavi

**Affiliations:** Kabul University of Medical Sciences, Kabul, Afghanistan; Afghanistan National Charity Organization for Special Diseases (ANCOSD), Kabul, Afghanistan; Kabul University of Medical Sciences, Kabul, Afghanistan; Faculty of Medicine, Kateb University, Kabul, Afghanistan; Kabul Infectious Disease Hospital, Kabul, Afghanistan; Medical Governance Research Institute, Tokyo, Japan; Department of Breast Surgery, Jyoban Hospital of Tokiwa Foundation, Fukushima, Japan; Department of Pharmaceutical Sciences, Nobel College, Affiliated to Pokhara University, Kathmandu, Nepal; Department of Nursing, Nobel College, Affiliated to Pokhara University, Kathmandu, Nepal; Medical Research Center, Kateb University, Kabul, Afghanistan; Afghanistan National Charity Organization for Special Diseases (ANCOSD), Kabul, Afghanistan

## 1 Introduction

Viral hepatitis is a liver disease caused by different viruses. These viruses can cause acute or chronic hepatitis and hepatocellular carcinoma (HCC). Viral hepatitis kills about 1.4 million people in the world annually and has received the status of the silent killer disease in Afghanistan by the World Health Organization (WHO) [1]. In addition, severe acute respiratory syndrome coronavirus-2 (SARS-COV2) virus has lethal effects on the liver besides other cells like pneumocytes. This virus binds to angiotensin-converting enzyme-2 (ACE-2) receptors on different cells, including hepatocytes, and causes their destruction and inflammation and deteriorates liver condition if already affected by hepatitis virus [2]. Additionally, some coronavirus disease 2019 (COVID-19) patients who visited hospitals had elevated alanine transaminase (ALT) and aspartate aminotransferase (AST) levels, the liver enzymes that often rise in case there is an insult to the hepatocytes [3].

Afghanistan is one of the least developed countries (LDCs), suffering from decades of war, and collapsed health system [4]. Hence, there is no complete data on any diseases in Afghanistan, including viral hepatitis. However, the database survey of viral hepatitis cases at Kabul Infectious Disease Hospital in 2019 and 2020 revealed 3945 recorded viral hepatitis cases in 2019 (2144 males and 1801 females), and 2706 (1509 males and 1197 females) recorded hepatitis patients in 2020. Most common viral hepatitis in Afghanistan is HBV transmitted by intravenous (IV) drug use, from mother to child (i.e., vertical transmission), from shared needle and cosmetic devices in barbershops, beauty salons and tattooing, and contaminated dental and surgical devices use [2]. Despite international humanitarian funding for fighting against the COVID-19 pandemic, the challenges in health care still prevail in the country [5], which in turn shadowed viral hepatitis patients [2].

Amidst the COVID pandemic, the Taliban re-captured Afghanistan's regime in August 2021. However, they again left the COVID and hepatitis patients at risk of developing severe lethal complications due to the country's lack of preparedness for effective and efficient health care delivery. [Kabul Infectious Disease Hospital].

## 2 COVID-added health and economic challenges

Epidemiological evidence revealed that a one-year delay in managing and controlling hepatitis (especially HCV) during the COVID-19 periods led to 906,000 undiagnosed cases and 746,000 deaths worldwide [2]. Total 200 million USD was spent to fight against the COVID-19 pandemic in Afghanistan [5]. Nevertheless, health challenges like shortage of beds in hospitals, lack of oxygen, essential medicines including antibiotics, analgesics, adrenaline, and vaccines and medical equipment still exist. Furthermore, western countries' political and economic sanctions against the Taliban regime have further deteriorated Afghanistan's existing failing health system. The medical staff has not received a salary for months [4,6,7]. During the Taliban government, 9 out of 37 COVID hospitals were shut [8], disrupting diagnosis and treatment of COVID-19, hepatitis, tuberculosis (TB), polio and many other diseases, especially in remote areas of Afghanistan [9].

Viral hepatitis poses a global burden on the health system, including Afghanistan, because of its costly screening, testing and management, complications, morbidity and mortality [10].

According to the Human Development Index (HDI), Afghanistan is the second LDC in the world [11] and is ranked 177th in the world [12]. In Kabul, unemployment was identified as an independent risk factor for HCV among younger injecting drug users (IDUs), and 90% of the commercial sex workers (CSWs) confessed that poverty was the driving motive to the sex trade [13].

Furthermore, there is a high prevalence of HBV and HCV in Afghan refugees out of 8 million living in neighboring countries like Pakistan and Iran, and they transmitted HBV and HCV to other Afghans while returning to Afghanistan [14].

Besides hepatitis, many patients with haemophilia, thalassemia, and hemodialysis also suffer from a lack of appropriate and timely medical care in Afghanistan, such as screened blood transfusion and deferoxamine (medicine for lowering iron levels) thalassemia treatment in Afghan hospitals [2].

## 3 Efforts to tackle health problems

Over the past two decades, the health system of Afghanistan has moderately improved [15]. State and private hospitals have been equipped with essential health services with national and international agencies to curb communicable diseases [10]. The Disease Early Warning System (DEWS), a surveillance system, was established in Afghanistan in 2006 and has expanded since then, reporting highly infectious diseases, including viral hepatitis [16]. The Health Management Information System (HMIS) provides monthly reports of health events from all public health facilities to the Ministry of Public Health (MoPH), but the system did not turn out to be successful [10].

Till 28 of July 2021, more than 46% of Afghan children received HBV vaccine with the support of the WHO. Still, uninterrupted access to screening and treatment facilities, with increasing public awareness and vaccination coverage, is required for effective control of viral hepatitis. Previously, WHO provided Afghanistan with pentavalent HBV vaccines (DPT-Hep B-Hib) in 2009, hepatitis B birth dose in 2014, and anti-HCV medicines in 2021 [1,2].

## 4 Conclusion

Viral hepatitis is a silent killer in Afghanistan, and low socio-economic conditions cause patients to be unaware of its prevention, complications and management.

COVID-19 pandemic drew global attention and urged every country to take the necessary actions to halt the spread of the virus. Unfortunately, despite being deadly and person-to-person transmissible, no particular actions have been taken to eliminate viral hepatitis from the country. Although viral hepatitis is as severe as the COVID-19 pandemic, neglecting the former disease may lead to dreadful complications and spread among the general population.

The emergence of the Taliban government further exacerbated Afghanistan's health system by restricting international donors' and humanitarian organizations' aid to the country. As a result, although patients with symptomatic hepatitis visited hospitals, hospitals lacked essential medicines, vaccines, and efficient health care delivery mechanisms.

## 5 Recommendations

• Enhancing epidemiological surveillance systems and capacity building of medical laboratories to provide reliable information for preventing and controlling viral hepatitis.
• Improving the national health system for efficient prevention and control of viral hepatitis through public awareness campaigns.
• Implementing safe injection and sterilized medical equipment use in invasive medical interventions at all national health facilities, particularly in rural areas with prevalent shared needle use.
• Strengthening vaccination coverage for a high-risk population and providing affordable hepatitis medication for the penniless.
• Encouraging national and international investments in hepatitis-related medical services.
• Ensuring viral hepatitis testing among susceptible pregnant women to prevent vertical transmission.
• Implementing policies, strategies, guidelines, and tools recommended by the WHO.

## Ethical approval

N/A.

## Please state any sources of funding for your research

None.

## Author contribution

All authors have contributed equally. Sayed Hamid Mousavi was the team leader and supervising the team.

## Consent

N/A.

## Registration of Research studies

1. Name of the registry: N/A.

2. Unique Identifying number or registration ID: N/A.

3. Hyperlink to your specific registration (must be publicly accessible and will be checked): N/A.

## Guarantor

Jawad Amini and Sayed Hamid Mousavi.

## Declaration of competing interest

There is no conflict of interest between all the authors.
